# Anticoronaviral Activity of the Natural Phloroglucinols, Dryocrassin ABBA and Filixic Acid ABA from the Rhizome of *Dryopteris crassirhizoma* by Targeting the Main Protease of SARS-CoV-2

**DOI:** 10.3390/pharmaceutics14020376

**Published:** 2022-02-08

**Authors:** Young-Hee Jin, Sangeun Jeon, Jihye Lee, Seungtaek Kim, Min Seong Jang, Chul Min Park, Jong Hwan Song, Hyoung Rae Kim, Sunoh Kwon

**Affiliations:** 1 KM Application Center, Korea Institute of Oriental Medicine, Daegu 41062, Korea; 2 Center for Convergent Research of Emerging Virus Infection, Korea Research Institute of Chemical Technology, Daejeon 34114, Korea; minseongjang@kitox.re.kr (M.S.J.); parkcm@krict.re.kr (C.M.P.); jhsong@krict.re.kr (J.H.S.); hyungrk@krict.re.kr (H.R.K.); 3 Zoonotic Virus Laboratory, Institut Pasteur Korea, Seongnam 13488, Korea; sangeun.jeon@ip-korea.org (S.J.); jihye.lee_01@ip-korea.org (J.L.); seungtaek.kim@ip-korea.org (S.K.); 4 Department of Non-Clinical Studies, Korea Institute of Toxicology, Daejeon 34114, Korea; 5 KM Convergence Research Division, Korea Institute of Oriental Medicine, Daejeon 34054, Korea

**Keywords:** *Dryopteris crassirhizoma*, dryocrassin ABBA, filixic acid ABA, coronavirus, SARS, MERS, COVID-19, antivirals, pharmacokinetics

## Abstract

The rhizome of *Dryopteris crassirhizoma* Nakai. (Dryopteridaceae) has been used in traditional medicine in East Asia and has recently been reported to have anticancer, anti-inflammation, and antibacterial activity as well as antiviral activity. Natural phloroglucinols from *D. crassirhizoma*, dryocrassin ABBA and filixic acid ABA were reported to inhibit influenza virus infection with an inhibitory activity on neuraminidase. In this study, we found that dryocrassin ABBA and filixic acid ABA have an inhibitory activity against the main protease of SARS-CoV-2. Therefore, dryocrassin ABBA and filixic acid ABA exhibited inhibitory activity against SARS-CoV-2 infection in Vero cells dose-dependently using the immunofluorescence-based antiviral assays. Moreover, these compounds inhibited SARS-CoV and MERS-CoV infection, suggesting their broad-spectrum anticoronaviral activity. In addition, a 5-day repeated-dose toxicity study of dryocrassin ABBA and filixic acid ABA suggested that an approximately lethal dose of these compounds in mice was >10 mg/kg. Pharmacokinetic studies of dryocrassin ABBA showed good microsomal stability, low hERG inhibition, and low CYP450 inhibition. In vivo pharmacokinetic properties of dryocrassin ABBA showed a long half-life (5.5–12.6 h) and high plasma exposure (AUC 19.3–65 μg·h/mL). Therefore, dryocrassin ABBA has therapeutic potential against emerging coronavirus infections, including COVID-19.

## 1. Introduction

In spite of 8.4 billion doses of coronavirus disease 2019 (COVID-19) vaccines, approximately 274 million confirmed cases of COVID-19 and 5.3 million deaths were reported as of December 2021 by the World Health Organization during the global pandemic [1]. Clinically effective therapeutics are urgently needed to minimize the cases of critical patients.

Emerging human coronaviruses, SARS-CoV-2 as well as SARS-CoV and MERS-CoV, belong to the *Coronaviridae* family and *Betacoronavirus* genus [2]. SARS-CoV-2 consists of a 30 kb positive-sense, single-stranded RNA and structural proteins, such as the nucleocapsid, envelope, membrane, and spike proteins. The SARS-CoV-2 genome shares approximately 79% genomic sequence with SARS-CoV and 50% with MERS-CoV [3]. Following SARS-CoV-2 cell entry into host cells by the spike protein binding to the angiotensin-converting enzyme 2 receptor, the viral RNA encodes two polypeptides, namely pp1a and pp1ab from two open reading frames, ORF1a and ORF1b, and four structural proteins [4]. The polyproteins are cleaved by the main protease (Mpro or 3CLpro) at 11 sites to generate numerous nonstructural proteins. This is an important step for viral replication [5].

Therefore, Mpro is considered an attractive target to inhibit viral replication [6,7]. Orally applicable viral protease inhibitors have been clinically used to treat hepatitis C virus and human immunodeficiency virus infections [8,9]. Mpro is highly conserved between SARS-CoV and SARS-CoV-2, with approximately 96% homology of amino acid sequence [10]; therefore, variant resistance is less expected. SARS-CoV-2 Mpro is a cysteine protease and has substrate specificity, which is absent in the human protease, expecting less toxicity in humans [11]. To develop state-of-the-art antivirals for treating COVID-19, high throughput screening for SARS-CoV-2 Mpro inhibitors has been executed from a natural compound library.

The rhizome of *Dryopteris crassirhizoma* Nakai. (Dryopteridaceae) has been used as traditional medicine in East Asia and was recently reported to have anticancer [12], anti-inflammatory [13], antibacterial [14], and anthelmintic [15] activity as well as antiviral activity against dengue virus [16]. It was reported that from >20 phloroglucinols isolated from *D. crassirhizoma*, dryocrassin ABBA and filixic acid ABA exhibited inhibitory activity against the neuraminidase of influenza virus and inhibited influenza virus infection [17].

In this study, we identified the SARS-CoV-2 Mpro inhibitory activity of natural phloroglucinols, dryocrassin ABBA and filixic acid ABA from the rhizome of *D. crassirhizoma,* before anticoronaviral activity against SARS-CoV, SARS-CoV-2, and MERS-CoV infections. In addition, we analyzed a 5-day repeated-dose toxicity study of these compounds and the pharmacokinetics of dryocrassin ABBA. Taken together, this study may provide the crucial information of potent natural compound inhibitors against the emerging coronavirus by targeting Mpro.

## 2. Materials and Methods

### 2.1. Test Compounds

Dryocrassin ABBA (PubChem ID 3082025, ≥98% purity) and filixic acid ABA (PubChem ID 15081408, ≥98% purity) were purchased from ChemFaces Biochemical Co. (Wuhan, China). Stock solutions (20 mM) were prepared in dimethyl sulfoxide (DMSO; Sigma-Aldrich, St. Louis, MO, USA). GC376 (BPS Bioscience, San Diego, CA, USA) were used as a positive control of Mpro activity assay in Mpro activity assay [18]. Lopinavir was purchased from SelleckChem (Houston, TX, USA) as a positive control [19] in immunofluorescence-based antiviral assay.

### 2.2. SARS-CoV-2 Mpro Activity Assay

SARS-CoV-2 Mpro activity was measured using the SARS-CoV-2 Mpro assay kit (BPS Bioscience, San Diego, CA, USA) according to the manufacturer’s instructions. The samples were evaluated in duplicated, serially diluted concentrations. The fluorescence intensity of low binding, black, 96-well microtiter plates was detected at an excitation wavelength of 360 nm and an emission wavelength of 460 nm using a microplate spectrophotometer (Bio-Tek, Winooski, VT, USA).

### 2.3. Cells and Viruses

Vero (ATCC^®^ CCL-81™) cells were purchased from the American Type Culture Collection (ATCC; Manassas, VA, USA) and maintained in Dulbecco’s modified Eagle’s medium (DMEM; Gibco, Carlsbad, CA, USA) with 10% fetal bovine serum (FBS; Gibco), and an antibiotic–antimycotic solution (Gibco) at 37 °C under 5% CO_2_. The Korea Disease Control and Prevention Agency kindly provided SARS-CoV-2 (βCoV/KOR/KCDC03/2020) and MERS-CoV (MERS-CoV/KOR/KNIH/002_05_2015) and Prof. J.S.M. Peiris from the University of Hong Kong kindly provided SARS-CoV (strain HK39849). Vero cells were used for virus propagation and plaque assays for titration. Experiments with SARS-CoV-2, SARS-CoV, and MERS-CoV were performed in a biosafety level-3 facility of the Institut Pasteur Korea (IP-K; Gyeonggi, Korea).

### 2.4. Immunofluorescence-Based Antiviral Assays

Vero cells (1.2 × 10^4^ cells) were cultured in DMEM with 1× antibiotic–antimycotic solution and 2% FBS in 384-well black culture plates. The serially diluted compounds and 0.0125 multiplicity of infection (MOI) SARS-CoV-2, 0.05 MOI SARS-CoV, or 0.0625 MOI MERS-CoV were added. At 24 h post-infection, the cells were fixed with 4% paraformaldehyde and stained with antibodies against the SARS-CoV-2 nucleocapsid protein, SARS-CoV spike protein, or MERS-CoV spike protein (Sino Biological Inc., Beijing, China), followed by goat antirabbit IgG secondary antibody and Hoechst 33342 (Thermo Fisher Scientific, Waltham, MA, USA). Images were analyzed using the Operetta^®^ High-Content Imaging System (20×; PerkinElmer, Inc., Waltham, MA, USA) and Image-Mining 3.0 plug-in software [20].

### 2.5. Five-Day Repeated-Dose Toxicity Study

Five-week-old C57BL/6 mice (male and female) were purchased from Orient Bio, Inc. (Gyeonggi, Korea) and housed at the Animal Care Facility of the Korea Institute of Toxicology (KIT; Daejeon, Korea) under 24 °C, 50% humidity, 12 h day/night cycle, and standard laboratory conditions. Mice were provided standard chow and drinking water and were acclimated for 7 days before the experiments. The experiments were approved by the Institutional Animal Care and Use Committee of KIT (approval number, KIT-B119020). The test compounds were intraperitoneally administrated at a dosage of 10 mg/kg/day in a solution of DMSO:polyethylene glycol (PEG) 400:distilled water (DW) (5:40:55) for 5 days. Each treatment group had five male and five female mice. The mice were observed and their body weights were measured daily for 6 days.

### 2.6. Liver Microsomal Metabolic Stability Assays

Liver microsome samples (0.5 mg protein/mL) from mouse, rat, and human (Corning, Glendale, AZ, USA) in PBS and the test compound (final concentration 1 μM) were mixed. Following the addition of the NADPH regenerating solution (Corning), the samples were incubated at 37 °C for 30 min. The reaction was stopped by the addition of ice-cold acetonitrile, and the samples were centrifuged at 4000 rpm for 15 min. The supernatant was analyzed using mass spectrometry with high-performance liquid chromatography (HPLC; Agilent Technologies, Santa Clara, CA, USA).

### 2.7. Human Ether-a-Go-Go-Related Gene (hERG) K^+^ Channel Activity Assays

The hERG K^+^ channel binding assay was performed with the HEK293 cell line expressing hERG using the automated planar patch clamp (PatchXpree 7000A) according to the manufacturer’s instructions.

### 2.8. Plasma Stability Assays

Animal plasma (Innovative Research, Inc., Novi, MI, USA) and test compounds (5 μM) were incubated at 37 °C for 4 h. To prevent the reaction, cold acetonitrile was added. After the samples were centrifuged, the supernatant was analyzed using mass spectrometry with HPLC (Agilent Technologies).

### 2.9. Cytochrome P-450 (CYP450) Enzyme Inhibition Assays

Human liver microsomes (0.2 mg protein/mL) were mixed with the test compound at 37 °C for 5 min. NADPH Regenerating solution (Corning) and specific substrates, namely phenacetin (CYP1A2, Sigma-Aldrich), tolbutamide (CYP2C9, Sigma-Aldrich), S-mephenytoin (CYP2C19, Corning), dextromethorphan (CYP2D6, Sigma-Aldrich), and sorafenib (CYP3A4, Santa Cruz Biotechnology, Inc., Dallas, TX, USA) were added and incubated at 37 °C for 30 min. Cold acetonitrile was added and the supernatant was analyzed using mass spectrometry with HPLC (Agilent Technologies).

### 2.10. Pharmacokinetic Studies in Mice

Seven-week-old male ICR mice were purchased from Nara Bio Co., Ltd. (Pyungtaek, Korea) and housed under 24 °C, 50% humidity, and 12 h day/night cycle conditions with access to standard chow and drinking water. Mice were acclimated for 1 week before the experiments. All animal procedures were approved by the KRICT Animal Care and Use Committee (ACUC approval number, DDP-7221). A total of 10 mg/kg of compound in a 5:40:55 ratio of DMSO:PEG400:DW was intraperitoneally or orally administered (n = 3). Blood samples were collected at 0.083–24 h after drug administration through the retro-orbital venous plexus and the plasma fraction was separated by centrifugation (15,000 rpm, 3 min). Following the addition of 9 volumes of acetonitrile to the plasma samples and centrifugation (13,000 rpm, 10 min at 4 °C), the supernatant was analyzed using mass spectrometry with HPLC (Agilent Technologies). Mean plasma concentration–time data were calculated using noncompartmental methods (Phoenix WinNonlin software version 6.4; Pharsight Corporation, Mountain View, CA, USA).

### 2.11. Statistical Analysis

Data were presented as the mean ± standard error of the mean of at least two independent experiments. Non-linear regression analysis of the half-maximal inhibitory concentration (IC_50_) was conducted using GraphPad Prism^®^ Software version 6.05 for Windows (GraphPad Software Inc., San Diego, CA, USA).

## 3. Results

### 3.1. SARS-CoV-2 Mpro Inhibitory Activity of Dryocrassin ABBA and Filixic Acid ABA

**Figure 1:**
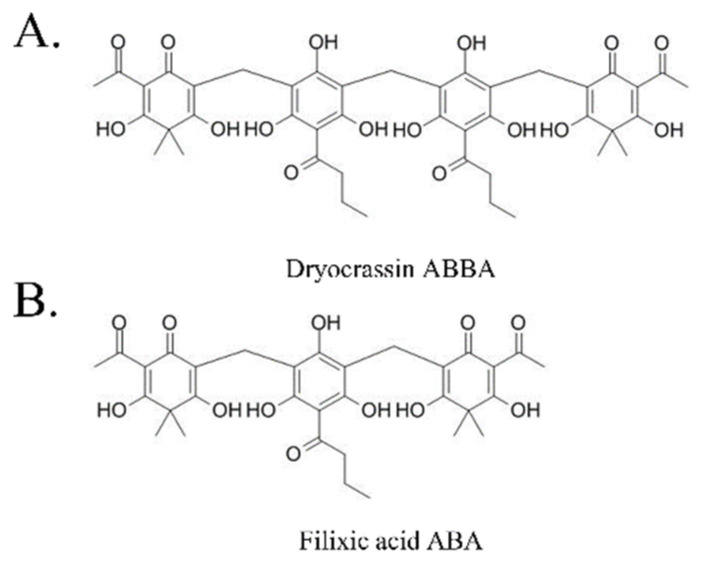
Chemical structure of dryocrassin ABBA (**A**) and filixic acid ABA (**B**).

**Figure 2:**
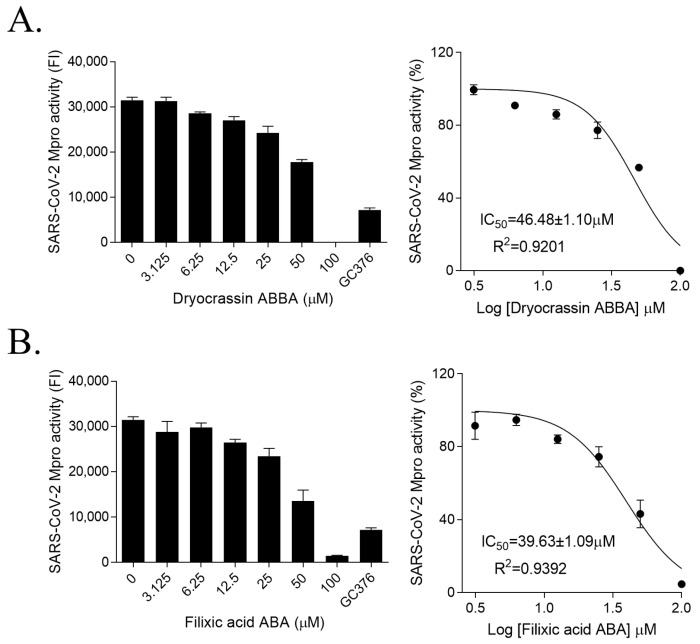
Inhibition of SARS-CoV-2 3CL activities of dryocrassin ABBA and filixic acid ABA (**A**,**B**). Dose-dependent inhibition of dryocrassin ABBA (**A**) and filixic acid ABA (**B**) in SARS-CoV-2 3CL activity analysis was shown with serially diluted compounds (3.125–100 μM). We used 100 μM GC376 as the positive control. IC_50_ values were calculated using non-linear regression analysis. The data are presented as the mean ± SEM of at least duplicate experiments.

To identify the inhibitor of SARS-CoV-2 Mpro, the natural compounds were screened using the SARS-CoV-2 Mpro assay kit. Among the screened compounds, dryocrassin ABBA and filixic acid ABA (Figure 1), which are the phloroglucinols from the rhizome of *D. crassirhizoma*, inhibited Mpro activity in a dose-dependent manner. Data showed that the IC_50_ values of dryocrassin ABBA and filixic acid ABA were 46.48 ± 1.1 μM and 39.63 ± 1.09 μM, respectively (Figure 2). These data suggest that dryocrassin ABBA and filixic acid ABA have a similar phloroglucinol structure and similar inhibitory activity against SARS-CoV-2 Mpro.

### 3.2. Anti-SARS-CoV-2 Activity of Dryocrassin ABBA and Filixic Acid ABA

**Figure 3:**
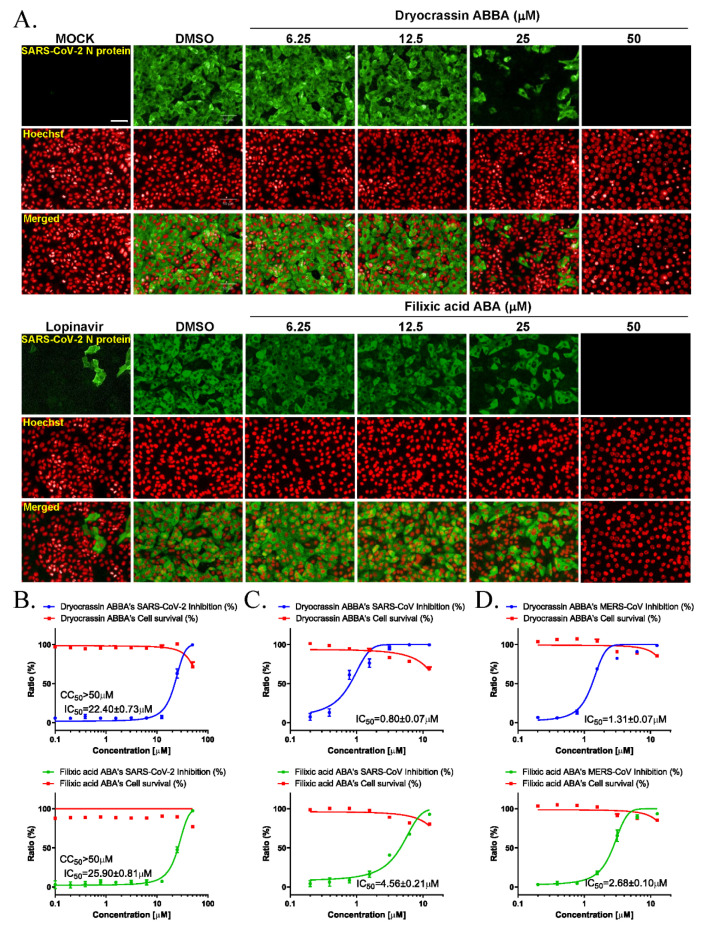
Anticoronaviral activities of dryocrassin ABBA and filixic acid ABA. (**A**) The confocal microscope images presented SARS-CoV-2 nucleocapsid (N) protein (green), cell nuclei (Hoechst, red) at the indicated concentrations of dryocrassin ABBA or filixic acid ABA, or 6.25 μM lopinavir after SARS-CoV-2 infection. Scale bar = 50 μm (**B**–**D**) Dose–response curve analysis using immunofluorescence staining was performed to determine the anti-SARS-COV-2 (**B**), SARS-CoV (**C**), or MERS-CoV (**D**) effect of dryocrassin ABBA (left panel) and filixic acid ABA (right panel) in virus-infected Vero cells. The inhibition of virus infection (%; blue circles for dryocrassin ABBA and green circles for filixic acid ABA) and cell viability (%; red squares) are indicated. IC_50_ values were calculated using non-linear regression analysis. The data are presented as the mean ± SEM of at least duplicate experiments.

We examined the anti-SARS-CoV-2 activity of dryocrassin ABBA and filixic acid ABA due to their inhibitory activity against SARS-CoV-2 Mpro. Immunofluorescence-based antiviral assays were performed in 0.0125 MOI SARS-CoV-2 infected Vero cells treated with a serially diluted concentration of these compounds by staining SARS-CoV-2 nucleocapsid antigen at 24 h after infection (Figure 3A). The IC_50_ value of each compound was calculated using non-linear regression analysis. Our data revealed that the IC_50_ values of dryocrassin ABBA and filixic acid ABA were 22.40 ± 0.73 μM and 25.90 ± 0.81 μM, respectively, with CC_50_ values of >50 μM (Figure 3B). These data suggest that these compounds had anti-SARS-CoV-2 activity, with similar IC_50_ values, through their inhibitory activity against SARS-CoV-2 Mpro.

### 3.3. Broad-Spectrum Anticoronaviral Activity of Dryocrassin ABBA and Filixic Acid ABA

To investigate whether these compounds have inhibitory activity against other coronaviruses, immunofluorescence-based antiviral assays were performed in 0.05 MOI SARS-CoV or 0.0625 MOI MERS-CoV-infected Vero cells treated with a serially diluted concentration of these compounds by detecting SARS-CoV spike protein or MERS-CoV spike protein, respectively, at 24 h post-infection (Figure S1). Our findings revealed that dryocrassin ABBA and filixic acid ABA inhibited SARS-CoV infection with an IC_50_ of 0.80 ± 0.07 μM and IC_50_ 4.56 ± 0.21 μM, respectively, (Figure 3C). In addition, dryocrassin ABBA and filixic acid ABA inhibited MERS-CoV infection with IC_50_ 1.31 ± 0.07 μM and IC_50_ 2.67 ± 0.10 μM, respectively (Figure 3D). These data suggest that dryocrassin ABBA and filixic acid ABA have broad-spectrum, anticoronaviral activity, and dryocrassin ABBA has better antiviral activity with a lower IC_50_ than filixic acid ABA.

### 3.4. Five-Day Repeated-Dose Toxicity of Dryocrassin ABBA and Filixic Acid ABA

**Figure 4:**
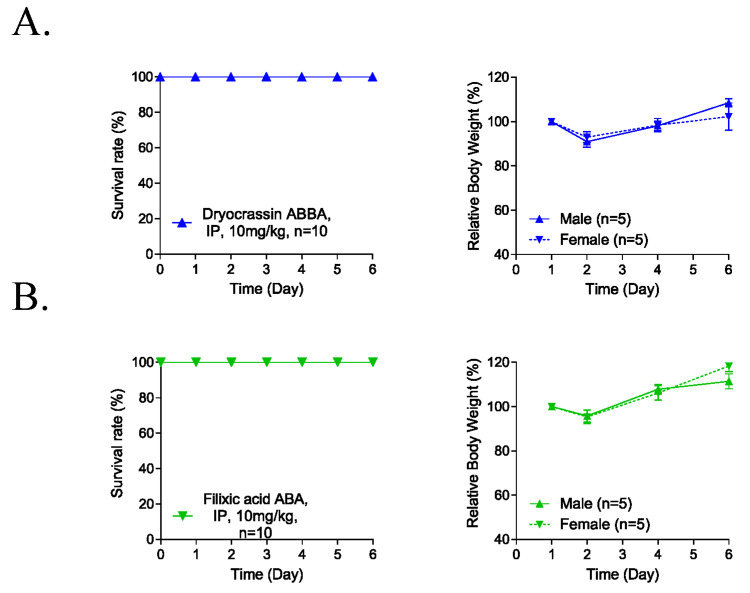
Five-day repeated-dose toxicity of dryocrassin ABBA and filixic acid ABA. (**A**,**B**) Survival rate (**left panel**) and body weight changes (**right panel**) in male (n = 5/group) and female (n = 5/group) mice intraperitoneally administrated dryocrassin ABBA (**A**) and filixic acid ABA (**B**) at 10 mg/kg/day for 5 days.

To evaluate the potential systemic toxicity of dryocrassin ABBA and filixic acid ABA, 5-day repeated-dose toxicity studies were conducted through IP administration of 10 mg/kg to male and female mice (each n = 5). No abnormal clinical signs and animal death were observed for 6 days after the IP administration of the compounds (Figure 4). Although the body weight was reduced at 2 day-post administration by 10%, it completely recovered 4 days post-administration of dryocrassin ABBA and filixic acid ABA. These data suggest that the approximately lethal dose of dryocrassin ABBA and filixic acid ABA in male and female mice was more than 10 mg/kg.

### 3.5. Pharmacokinetics of Dryocrassin ABBA

**Figure 5:**
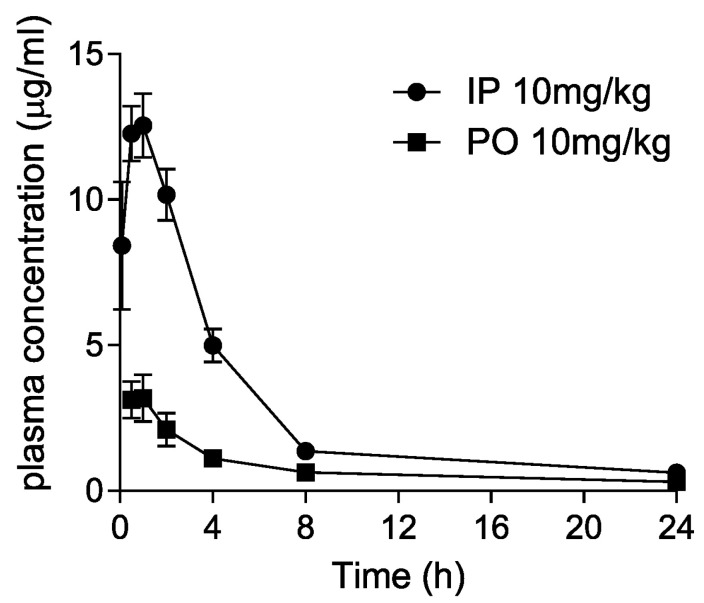
Pharmacokinetic plasma concentration–time curves following intraperitoneal (IP) and oral (PO) administration of 10 mg/kg dryocrassin ABBA in mice.

Dryocrassin ABBA was selected to further investigate the pharmacological features such as liver microsomal stability, hERG inhibition, plasma stability, and CYP450 inhibition (Table 1). Dryocrassin ABBA was examined against mouse, rat, and human liver microsomes. It was well tolerated in mouse, rat, and human liver microsomes, with more than 68% remaining after 30 min incubation. In the hERG patch clamp assay, the IC_50_ value of dryocrassin ABBA on hERG potassium channel was less than 50 μM and the inhibition percentage was 7.27% at 10 μM, suggesting the cardiac safety of dryocrassin ABBA. Plasma stability of dryocrassin ABBA was lower in the human model (29.67% ± 8.37% remaining) than in the rat model (67% ± 7.29% remaining). The CYP450 inhibition assay data revealed that the IC_50_ values of dryocrassin ABBA were 8.33–16.1 μM on isozymes, except for 0.4 μM IC_50_ on 2C9 isozyme. Finally, we analyzed the in vivo pharmacokinetic (PK) properties of 10 mg/kg dryocrassin ABBA in mice (n = 3) by intraperitoneal (IP) and oral (PO) administration (Figure 5 and Table 2). The half-life of the compound via IP was 5.5 h and the AUC value was 65 μg·h/mL. In the case of PO administration, the half-life of the compound was 12.6 h and the C_max_ value was 3.64 μg/mL, reflecting 19.3 μg·h/mL AUC. These data suggest that dryocrassin ABBA is an acceptable candidate as a COVID-19 therapeutic drug.

## 4. Discussion

The rhizome of *D. crassirhizoma* is used as traditional medicine against inflammation. The rhizome of *D. crassirhizoma* is one of the main components of the Lianhua-Qingwen formula, which is composed of 11 herbal medicines traditionally used for heat-clearing and detoxifying, recently used for the prevention and treatment of viral influenza [21,22], SARS, and COVID-19 in China [23,24].

Among the constituents derived from the rhizome of *D. crassirhizoma*, kaempferol glycosides have been known to have inhibitory activity on human immunodeficiency virus reverse transcriptase [25]; hopane-type triterpenes are also reported to have an inhibitory activity on human immunodeficiency virus protease [26]. Flavaspidic acid AB compound from *D. crassirhizoma* inhibited the replication of porcine reproductive and respiratory syndrome virus by inhibiting virus internalization and inducing antiviral cytokines [27]. Moreover, phloroglucinols from *D. crassirhizoma*, dryocrassin ABBA and filixic acid ABA exhibited inhibitory effects on the NA of H5N1 influenza virus with an IC_50_ of 18.59 ± 4.53 and 29.57 ± 2.48 μM, respectively; however, only dryocrassin ABBA exhibited anti-influenza virus activity with an IC_50_ of 16.5 μM and filixic acid ABA with <50% at 100 μM [17].

Our findings revealed that two phloroglucinols isolated from *D. crassirhizoma*, dryocrassin ABBA and filixic acid ABA, have dose-dependent inhibitory activity against the Mpro of SARS-CoV-2 virus, which may result in preventing the cleavage of coronaviral polypeptides, pp1a and pp1ab, into nonstructural proteins for viral replication [5]. Ultimately, these compounds inhibited SARS-CoV-2 virus infection as well as other coronavirus infections, including SARS-CoV and MERS-CoV due to the highly conserved homology among coronaviral Mpros [10]. These data suggest that it is possible to develop dryocrassin ABBA and filixic acid ABA as a potent broad-spectrum anticoronaviral therapeutic drug, including COVID-19 by direct acting antivirals targeting the Mpro.

For drug development, a 5-day repeated-dose toxicity study of dryocrassin ABBA and filixic acid ABA helped identify that the approximately lethal dose of dryocrassin ABBA and filixic acid ABA in mice was >10 mg/kg. Pharmacological assays, such as liver microsomal stability assay, hERG inhibition assay, plasma stability assay, and the CYP450 inhibition assay of dryocrassin ABBA suggested good stability, low hERG channel inhibition, and low CYP450 inhibition, except for 2C9 isozyme activity. Investigations of the in vivo pharmacokinetic properties of dryocrassin ABBA showed a long half-life and high plasma exposure. Pharmacodynamic study and in vivo proof of concept would be necessary in future study.

## 5. Conclusions

We found that natural phloroglucinols, dryocrassin ABBA, and filixic acid ABA, play a role in the inhibition of SARS-CoV-2 infection by inhibiting the Mpro of SARS-CoV-2 as well as have broad-spectrum anti-emerging coronaviral activity. Furthermore, the preclinical studies on the toxicity and pharmacokinetics of dryocrassin ABBA suggested that dryocrassin ABBA is a promising therapeutic candidate for the development of anticoronaviral drugs, including those against COVID-19.

## Figures and Tables

**Table 1:** Microsomal stability (MS), hERG inhibition, plasma stability (5 μM), and cytochrome P450 (CYP450) inhibition of dryocrassin ABBA.

MS (%) (Remaining after 30 min)			hERG Inhibition	Plasma Stability (% Remaining)		CYP450 Inhibition (IC_50_, μM)				
Mice	Rat	Hum		Hum	Rat	1A2	2C9	2C19	2D6	3A4
68.3 ± 2.1	>99	>99	7.27% (10 μM) IC_50_ > 50 μM	29.67 ± 8.37	67.00 ± 7.29	14.7	0.4	16.1	13.5	8.33

**Table 2:** Mouse pharmacokinetic study of dryocrassin ABBA.

Parameters (n = 3)	IP (10 mg/kg)	PO (10 mg/kg)
T_max_ (h)	1.17 ± 0.76	0.67 ± 0.29
C_max_ (μg/mL)	12.81 ± 1.79	3.64 ± 0.57
T_1/2_ (h)	5.5 ± 0.56	12.6 ± 3.76
AUC (μg∙h/mL)	65.96 ± 8.63	19.3 ± 1.42

## Data Availability

The data presented in this study are available in this article.

## Supplementary Materials

The following are available online at https://www.mdpi.com/article/10.3390/pharmaceutics14020376/s1, Figure S1: The confocal microscope images showing the SARS-CoV spike (S) protein (green) (A), MERS-CoV spike (S) protein (green) (B), and cell nuclei (Hoechst, red) at the indicated concentration of each compound or 25 μM lopinavir after SARS-CoV (A) or MERS-CoV (B) infection.

## Abbreviations

3CLpro	3C-like protease
CC_50_	Half-maximal cytotoxic concentration
CoV	Coronavirus
COVID-19	Coronavirus disease 2019
CYP450	Cytochrome P-450
hERG	Human ether-a-go-go-related gene
IC_50_	Half-maximal inhibitory concentration
MERS-CoV	Middle East respiratory syndrome coronavirus
Mpro	Main protease
NA	Neuraminidase
ORF	Open reading frame
SARS-CoV	Severe acute respiratory syndrome coronavirus
SARS-CoV-2	Severe acute respiratory syndrome coronavirus 2
