# Supplementary material for: Anticoronaviral Activity of the Natural Phloroglucinols, Dryocrassin ABBA and Filixic Acid ABA from the Rhizome of Dryopteris crassirhizoma by Targeting the Main Protease of SARS-CoV-2
Source: Pharmaceutics. 2022 Feb 8;14(2):376. doi: 10.3390/pharmaceutics14020376 (PMC8879496; doi:10.3390/pharmaceutics14020376)
Supplement: Supplementary file 1 [file pharmaceutics-14-00376-s001.zip › pharmaceutics-1549764-supplementary.pdf]

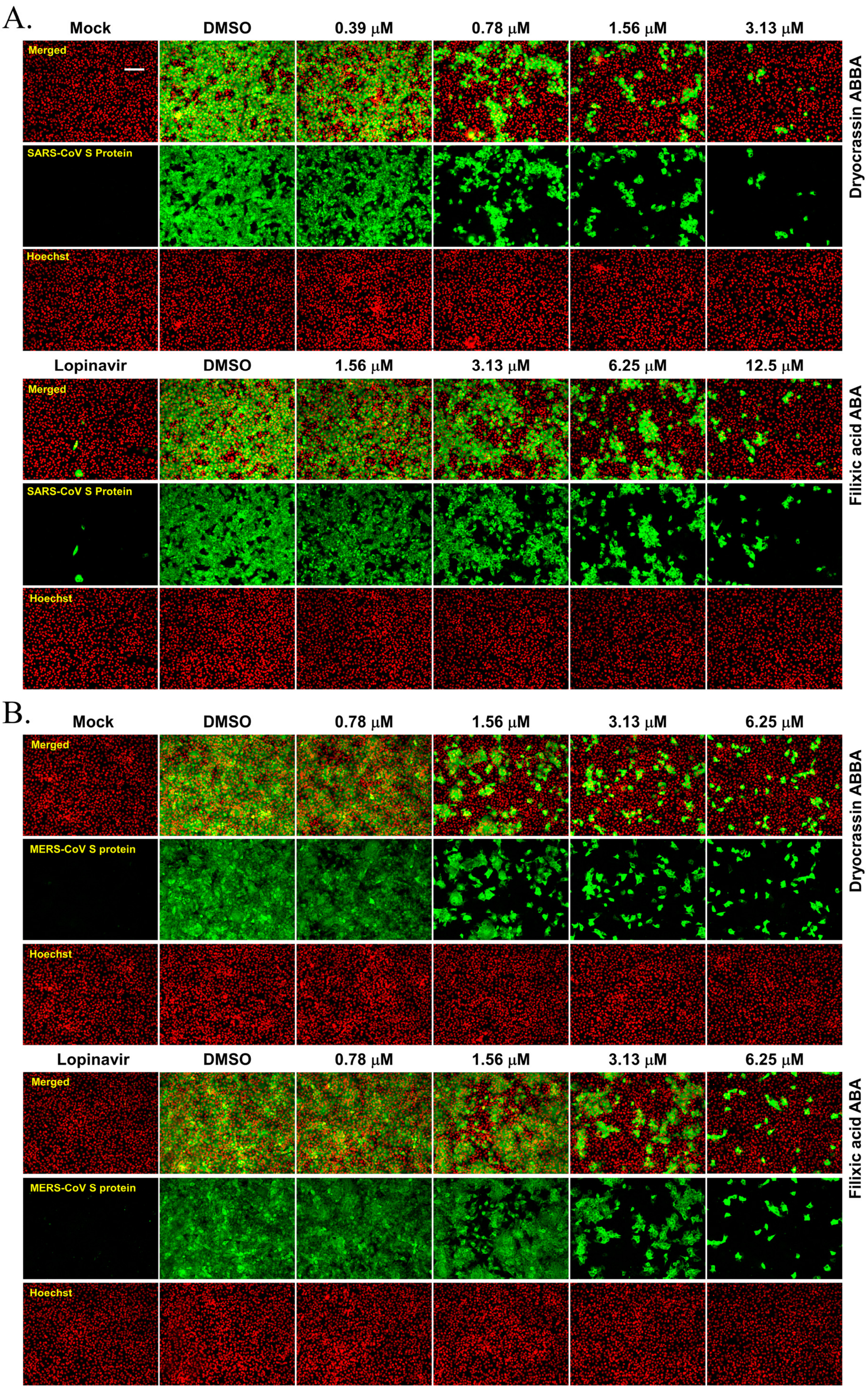

Figure S1. The confocal microscope images showed SARS-CoV spike (S) protein (green) (**A**), MERS-CoV spike (S) protein (green) (**B**), and cell nuclei (Hoechst, red) at the indicated concentration of each compounds or 25 μM lopinavir after SARS-CoV (**A**) or MERS-CoV (**B**) infection. Scale bar = 100 μm
